# How information systems should support the information needs of general dentists in clinical settings: suggestions from a qualitative study

**DOI:** 10.1186/1472-6947-10-7

**Published:** 2010-02-02

**Authors:** Mei Song, Heiko Spallek, Deborah Polk, Titus Schleyer, Teena Wali

**Affiliations:** 1 Center for Dental Informatics in the Department of Dental Public Health and Information Management, School of Dental Medicine, University of Pittsburgh, 3501 Terrace Street, Pittsburgh, PA 15261, USA; 2 Department of Dental Public Health and Information Management, School of Dental Medicine, University of Pittsburgh, 3501 Terrace Street, Pittsburgh, PA 15261, USA

## Abstract

**Background:**

A major challenge in designing useful clinical information systems in dentistry is to incorporate clinical evidence based on dentists' information needs and then integrate the system seamlessly into the complex clinical workflow. However, little is known about the actual information needs of dentists during treatment sessions. The purpose of this study is to identify general dentists' information needs and the information sources they use to meet those needs in clinical settings so as to inform the design of dental information systems.

**Methods:**

A semi-structured interview was conducted with a convenience sample of 18 general dentists in the Pittsburgh area during clinical hours. One hundred and five patient cases were reported by these dentists. Interview transcripts were coded and analyzed using thematic analysis with a constant comparative method to identify categories and themes regarding information needs and information source use patterns.

**Results:**

Two top-level categories of information needs were identified: foreground and background information needs. To meet these needs, dentists used four types of information sources: clinical information/tasks, administrative tasks, patient education and professional development. Major themes of dentists' unmet information needs include: (1) timely access to information on various subjects; (2) better visual representations of dental problems; (3) access to patient-specific evidence-based information; and (4) accurate, complete and consistent documentation of patient records. Resource use patterns include: (1) dentists' information needs matched information source use; (2) little use of electronic sources took place during treatment; (3) source use depended on the nature and complexity of the dental problems; and (4) dentists routinely practiced cross-referencing to verify patient information.

**Conclusions:**

Dentists have various information needs at the point of care. Among them, the needs for better visual representation and patient-specific evidence-based information are mostly unmet. While patient records and support staff remain the most used information sources, electronic sources other than electronic dental records (EDR) are rarely utilized during patient visits. For future development of dental information or clinical decision-support systems, developers should consider integrating high-quality, up-to-date clinical evidence into comprehensive and easily accessible EDRs as well as supporting dentists' resource use patterns as identified in the study.

## Background

Physicians and dentists have diverse information needs when making informed diagnoses and treatment decisions. While medical knowledge continues to grow at a steady pace [1], clinicians are spending less time with patients [2], leading to lower patient satisfaction [3]. As a result, identifying information clinicians need at the point of care and accurately and efficiently providing it have become issues of critical importance.

The majority of studies on clinicians' information needs have focused on primary or ambulatory care settings. One approach was to identify the differences in information needs with respect to characteristics of clinicians, such as specialty and practice setting [4-7]. Another was to examine the nature of clinical questions posed by physicians and to build taxonomies of questions to identify the most common generic types [8,9]. More recently, the use of case-specific questions has been advocated as a more valid way to understand physicians' information needs at the point of care, with distinct characteristics exhibited by physicians according to search context and case scenarios [10]. Only a very small number of studies have looked at dentists' information needs. For example, Strother, Lancaster and Gardiner [11] surveyed 500 dentists and found that they needed information on new techniques in dentistry, followed by information on products and equipment, the temporomandibular joint, practice management, and medical complications. Duxbury and Leach [12] identified drug interactions and adverse reactions, precautions and dosage as top inquiries related to drug use.

Research on clinicians' use of information sources has yielded less variation than research on their information needs over the years [10]. In 1997, Haug's meta-analysis indicated that physicians most frequently found medical information in desk references (journals and books) and through consultations with colleagues [13]. In recent years, with the enormous growth in electronic resources, the use of online evidence sources has become an interest of research. For instance, studies in Australia found that general practitioners used an online evidence retrieval system during consultation with patients, and reported improved quality of care [14]; however, usage rate dropped significantly after initial introduction of the system in a 12-month study [15]. In dentistry, professional colleagues, personal journal collections and continuing education were reported as the top three information sources by dentists [11]. In a 1991 study, medical reference books were listed as the number one source for drug information for dentists, followed by colleagues and dental reference books [16]. Factors found to influence clinicians' choice of information resources included specialty, type and locus of practice, experience, age, and work roles and tasks [4,5,17-19]. While approximately 25% of all general dentists in the United States used chairside computers as of 2006 [20], little is known as to whether and how dentists use online sources in clinical practice.

To meet clinicians' information needs more efficiently during clinical work, various strategies have been proposed, developed and implemented. One solution is to provide them with evidence-based clinical guidelines in an easily accessible format and another is to support their diagnosis and treatment with health information systems/tools, such as clinical decisions support systems (CDSS) that are able to manage large amounts of information and draw correct inferences based on specialty-level knowledge and patient-specific data. The development of health information systems, CDSS in particular, has been active in medicine and dentistry in the last two decades [21]. In dentistry, White [22] reviewed over 30 decision support applications and classified them into seven areas of dentistry (dental emergencies, orofacial pain, oral medicine, oral radiology, orthodontics, pulpal diagnosis and removable prosthodontics). Notable examples of such support applications include the Diagnostic Aid Resource Tool (DART), which assists in the diagnosis and management of oral diseases in the head and neck [23]; ORAD, which advises clinicians on the interpretation of radiographic lesions [24]; and a computerized approach which is used for caries management [25].

Despite the many benefits promised by these systems, few have helped to improve clinician performance, and even fewer have influenced patient outcomes [26-28]; thus, their adoption in medicine, particularly in dentistry, has been slow and limited [29], with little impact on clinical practice [30]. One critical reason, as Wears and Berg point out, is that the designers of these systems often fail to view the clinical workplace as a complex sociotechnical system, and therefore misunderstand that the nature of clinical work is collaborative, distributed, interpretative, interruptive and reactive [31]. This often makes the designed systems very difficult to use. Specifically, important inhibiting factors include the systems' poor integration into dental workflow; inability to provide an integrated view of patient data; lack of best clinical evidence in the knowledge base; limited understanding of dentists' information needs and decision-making processes; and practitioners' skepticisms about the value and feasibility of these systems [29,32,33].

The purpose of this study was to identify the information needs of general dentists at the point of care and their use of information sources. Through thematic analysis, we categorized their information needs and source use and described themes related to the information needs and source use patterns that emerged from the analyses. The results are expected to inform the development of methods, strategies and information systems to meet dentists' information needs and thus support their diagnosis and treatment in more efficient and effective ways.

## Methods

### Procedure

We contacted 159 general dentists within the zip codes of the Pittsburgh area from a list of 228 American Dental Assocation (ADA) members by telephone or email to invite them to participate in our study (specialists not included in our sample). No incentives were involved in the recruitment. Due to the difficulty in recruiting busy practitioners, we obtained a convenience sample of 18 general dentists. We conducted a semi-structured interview with each participant after visiting their practice for half a day during clinic hours. An interview protocol was developed and pilot tested with several dental faculty and dentists at the University of Pittsburgh (see Additional file 1). The interview focused on dentists' information needs arising during treatment sessions, the sources they used to acquire the information, and any information that would have been helpful to them but was unavailable at the time. All participating dentists were interviewed between October 2007 and April 2008.

On the interview day, one researcher [TW] visited the dental office for half a day. Following each patient visit, the researcher interviewed the dentist and audio taped the session without any patient identifiers mentioned on the tape. The demographic information of each dentist was also collected. The interviews were subsequently transcribed verbatim with all possible identifiers removed. This study was approved by the University of Pittsburgh Institutional Review Board (#07080148).

### Coding and analysis

We applied an inductive analysis approach, grounded in the respondents' answers without any preconceived assumptions on the part of the researchers. Specifically, we used the thematic analysis method to analyze the interview transcripts. Thematic analysis is a method for identifying, analyzing and reporting patterns (themes) in data. In contrast to other qualitative methods, such as grounded theory or narrative analysis, thematic analysis is not wedded to any pre-existing theoretical framework; thus it can be used within different theoretical frameworks as either a realist or constructionist method [34].

We conducted the analysis following the methods of Braun and Clarke [34]. Two trained analysts (MS, HS) open-coded the transcripts independently to identify categories regarding dentists' information needs and use of information sources, with special attention to their unmet information needs. They then compared the codes and reached consensus on coding discrepancies through discussion. Any code identified was matched with data extracts that demonstrated that code. All data extracts were then collated together within each code. One researcher (MS) then related data extracts within and across categories to identify potential themes, and the whole team systematically reviewed these themes to make sure that they worked in relation to both the coded extracts and the entire data set.

## Results

In this section, we first describe the demographics of the participants. Then we present the categories of information needs and source use found, followed by the themes that emerged from the thematic analysis.

### Demographics

Eighteen dentists completed the study (13 males and 5 females). They were on average 47 years old (range: 30-64 years) and had been practicing for 20.7 years (range: 2-32 years). Twelve of them graduated from the School of Dental Medicine at the University of Pittsburgh and six from four other dental schools. Among these dentists, fourteen had professional memberships, such as in the American Dental Association and Pennsylvania Dental Association. Twelve offices used a practice management system for patient scheduling and insurance purposes. Four offices had chairside computers with Internet access in the operatories (a small room in a dental office for performing treatment, typically equipped with a fixed patient chair and various other equipment, such as a delivery tray with dental hand pieces, an overhead dental light, high volume suction, and x-ray). Eight dentists used emails for clinical purposes such as patient referrals or consults.

### Information needs of general dentists

Dentists' information needs varied by nature and number depending on the clinical situation and patient characteristics. For the 105 patient cases, dentists reported a total of 320 information needs (256 met needs and 64 unmet needs), averaging 3.1 per patient (range: 1 to 7). Two top-level categories of information needs with several subcategories emerged from the coding: foreground information needs and background information needs.

#### Foreground information needs

Information needs in this category refer to the questions or concerns a dentist had regarding the medical or technical aspects of the current dental disease or condition at hand, such as the symptoms, treatment options/procedures, effect of treatment and clinical evidence. In all 105 cases, dentists had at least one question of this kind. Most commonly, they were concerned about chief complaint and symptoms, assessment of a problem, treatment options and procedures, clinical evidence regarding a dental problem, and responses to treatment. For example, in 55 cases, dentists inquired explicitly about the patient's chief complaint and symptoms; and in 41 cases, they needed information on different aspects of a dental problem, such as the location and size of cavities, measurement of root canals, or depth of pockets, in order to assess its scale and severity.

#### Background information needs

Dentists also inquired about issues beyond the medical or biological aspects of a dental problem. These information needs, such as medical history, dental health behaviors and dental insurance, though at the background of the current problem, are indispensable in helping dentists to reason about possible causes of the problem to make well-informed diagnoses and joint treatment decisions with patients. In 33 cases, dentists looked for information such as chronic diseases, medications and allergic histories in the medical and health history to help assess the dental problem and decide on drug use. For some procedures, dentists were required to get authorization from insurance companies prior to the therapy; therefore, in 17 cases, information needs arose regarding patient-specific coverage, billing and payment issues. For another 11 patients, when dentists had special concerns about dental materials and costs of treatments, dentists asked patients for their input on the choice of treatment to make joint decisions. Table 1 summarizes all the information needs dentists reported during the 105 clinical sessions and reports the percentages of cases in which the needs were met or unmet.

**Table 1:** Information needs of general dentists during clinical sessions in the study (n = 105 patient cases)

**Foreground information needs**	**Cases with needs**	**Cases with needs met**	**Cases with needs unmet**	**Background information needs**	**Cases with needs**	**Cases with needs met**	**Cases with needs unmet**
	
Chief complaint & symptoms	55	55 (100%)	0 (0%)	Medical & health history	36	33 (92%)	3 (8%)
	
Treatment options & procedures	50	44 (88%)	6 (12%)	Dental insurance coverage	17	12 (71%)	5 (29%)
	
Visual representation of problems	43	31 (72%)	12 (28%)	Patient decision on treatment	11	10 (91%)	1 (9%)
	
Assessment of problems	41	36 (88%)	5 (12%)	Time of dental visit	8	8 (100%)	0 (0%)
	
Effect of treatment	15	12 (80%)	3 (20%)	Dental health behaviors	8	7 (87.5%)	1 (22.5%)
	
Clinical evidence	9	0 (0%)	9 (100%)	Access to resources	8	4 (50%)	4 (50%)

### Themes regarding dentists' information needs

#### Dentists needed timely access to information on various subjects

Due to the fast-paced schedule in a dental office, quick access to clinical and other information was considered essential to patient diagnosis and treatment. Information that dentists had difficulty obtaining in a timely manner included data previously entered in paper-based patient records, tooth conditions shown in radiographs taken at other dental offices, and insurance coverage and authorization information. This lack of timely access was reported to have delayed dentists' diagnosis, treatment and patient education. For instance, Dentist 17 was scheduled to take impressions of a patient for a denture but did not have access to the panoramic x-ray taken at the last visit as it had been sent to an oral surgeon. Commenting on its absence, he said, *"I had reviewed it and made a recommended treatment plan for the lower... but that would have been helpful to be able to show the patient the specific teeth I was concerned about."*

The slow processing time and, sometimes, the unwillingness of insurance companies to share patient information were viewed as important limiting factors by some dentists. Dentist 9 needed the payment history of a patient from his insurance company to calculate the cost for the current treatment. The company first refused to share any information and later gave him incorrect information, resulting in deep frustration and even anger on the part of the dentist.

#### Dentists preferred better visual representations of dental problems

Dentists in the study wished that better representation of dental problems were available to them in addition to the radiographs and intraoral pictures they routinely use to support clinical decision making. They preferred the presentation in various formats, such as a mounted case, pictures, 2D digital images and 3D visualization, with the best image quality. While emerging health IT tools have the capacity to show detailed tooth conditions in color and even bone and tooth structures in 3D visualization, dentists' needs were not met due to lack of access to equipment or computer malfunctions. When treating a patient with occlusal caries and a crown fracture, Dentist 18 wished he had instant digital images to show the patient, who requested to see how the restoration would look aesthetically. *"Having digital imaging capabilities, not x-rays in this case, but digital from the stand point of color photography so that I would be able to magnify and put onto a flat panel monitor, would have been very helpful," *he said.

#### Dentists desired access to patient-specific evidence-based information

In a standard evidence-based approach, dentists need to combine information based on clinical evidence with their skills and expertise as well as the patient's needs and preferences [35]. Therefore, having access to patient-specific evidence-based information at the right time can help dentists make informed decisions, predict the effectiveness and outcome of treatments, and educate patients about their choices.

In this study, the evidence-based information dentists needed but failed to obtain were primarily concerned with the longevity and success rate of crowns and bridges, the durability of devices such as night guards and the probability that a patient might develop a certain dental problem in the future. For example, Dentist 3 had a patient for a crown insertion, and the biggest question he had was how long the crown would last. Having seen crowns that lasted 20-25 years and ones that failed in one year, he wished he could have had *'statistics about the actual longevity of crowns and bridges." *When treating a child patient, the ability to predict what problems s/he will develop when growing up is essential from a preventive point of view. This was reflected in the case of a 10-year-old patient of Dentist 5 who was concerned about the child growing up to be a *"hard treat" *for an orthodontist. He expressed the wish that *"there was something I could access and look it up"*, making the parents aware of expected outcomes based on age and risk factors.

#### Documentation of patient records should be accurate, complete and consistent

Some dentists had to delay treatment due to inaccurate, incomplete or inconsistent recording of patient information in various places. This problem was sometimes attributed to the existence of dual systems--the paper-based and electronic patient records--which created extra work in verifying information. This was true for Dentist 1, who had inconsistent information on a treatment plan for a patient in three places, a routing slip (a slip of paper attached to the paper-based patient chart that specifies a route for a patient and his/her chart to circulate through the dental office), computer and the actual chart. Other times a more detailed description of a tooth problem was needed to avoid confusion. For example, a patient of Dentist 17 needed restorations on #7 distal, facial and incisal; however, treatment on the facial surface was missing in the progress notes, and it took extra time to locate and verify at several places.

### Use of information sources of general dentists

To get the information they needed, dentists in the study relied on various sources. These fell into four categories based on their functions: sources for clinical information/tasks, sources for administrative tasks, sources for patient education, and sources for professional development. While the first two were routinely used by all dentists for most patient cases, six dentists reported using patient education sources for specific patient cases and three discussed patient cases with colleagues in study groups. For the 105 cases, dentists used a total of 296 sources, with an average of 2.8 sources per patient (range: 1 to 6). Each component of the patient record, such as the medical history or radiographs, was considered as a different source in the analysis.

The most important clinical source was the patient record, comprising several key components: medical history, periodontal and hard tissue charts, radiographs, treatment plan, and progress notes. The second most used source was support staff, including hygienists, dental assistants and people in the lab, followed by patients. Dentists used different sources to educate patients, from showing them dental problems and explaining treatment options, to teaching them how to brush correctly. Besides the commonly used pictures and flip charts showing tooth structure and jaw joints and muscles, three dentists used education software, such as Casey (Patterson Companies, Inc, St. Paul, MN), to give patients an interactive and more memorable experience. Sources for administrative tasks were mainly interpersonal, with the front desk handling scheduling, and billing and insurance checking tasks shared by the front desk, office manager and occasionally the chairside assistant. After clinical hours, some dentists tried to keep up with the development of the profession, usually through study clubs/groups, meetings and continuing education courses and events.

### Themes regarding dentists' information needs

#### Dentists' information needs matched information source use in general

Dentists in this study obtained most clinical information from the patient record. As different sections of the record are designated for different information, dentists usually went directly to a specific section for what they needed, such as patient medication from medical history and previous treatments from the treatment plan or progress notes. A clear division of responsibilities was usually enforced in these dental offices. For instance, a dentist confirmed with an assistant about the reason for a patient visit, but asked a hygienist to report problems after a cleaning. Similarly, scheduling and insurance checking was mostly handled by the front desk, though occasionally the office manager or assistant got involved in insurance issues. One exception was observed in Dentist 18's office, where the hygienist was much more involved in discussing diagnosis and treatment than in any other office in the study.

#### Dentists used few electronic sources/tools to meet information needs during treatment

Twelve of the participating dentists used a practice management system, but for little than scheduling and billing purposes. Three dentists reported using electronic patient records, but simultaneously with a paper chart for fear that the computer record was not accurate or detailed enough. While eight dentists had an Internet connection in the operatories, none of them went online for any patient during our visit. The rare use of electronic sources/tools was partly due to the dentists' busy schedules and partly due to the unavailability of or difficulty in finding evidence-based information. Two dentists reported using electronic sources sometimes before or after patient visits. For example, Dentist 8 used *"the computer in my office" *to verify and follow up on information provided by patients about a disease or medication he was unfamiliar with. To save time, Dentist 4 preferred doing his *"homework," *searching for diseases and medications online, before seeing patients the next day. If unable to get any information during the appointment, he would rather *"reappoint the patient if it is not an emergency situation" *than continue the treatment and risk making errors.

#### Source use depended on the nature and complexity of the dental problem at hand

Dentists in general used fewer sources to treat simple cases, such as new patient examinations, but checked multiple sources and sometimes consulted with specialists about diagnosis and treatment for relatively complex cases, such as a crown or root canal treatment. For a new patient examination, they normally looked at the medical history and took bitewings or other necessary x-rays. When undertaking complex cases requiring multiple treatment sessions, they referred to various sections of the record, such as radiographs, progress notes, medical history, hard tissue charts and insurance coverage, to gather different information. They also interacted more with patients to explain the details of a procedure and asked for patient feedback.

Despite the pattern above, in four patient cases, dentists used fewer sources than they normally would to treat the patients. One situation was when they still had a vivid memory of a patient. Another situation was when a veteran dentist, relying on years of acquired knowledge and skills, felt that he needed little additional information for certain cases. For example, when asked if he looked at anything else other than scanning the progress notes to decide on a partial fitting treatment for a patient, Dentist 7 said, *"I know the next treatment that needs to be done, because I've been doing it for 32 years, not because it is written there on a piece of paper. A lot of this information I don't need a source of information, just like when you have breakfast and you reach in the drawer to get a spoon to eat your cereal, you didn't have to go to some source and find out spoons are good for eating cereal. The source of information is just in my head."*

#### Dentists routinely practiced cross-referencing to verify the validity and accuracy of information

Dentists in the study regularly checked multiple sources to ensure that critical clinical information (such as tooth number or root canal measurements) were correct, though the way they did this varied due to practice style. While Dentist 5 always started with the patient chart and then double-checked with the patient, Dentist 2 preferred getting the patient's story before verifying it with his record. The three key sources for their information triangulation were the schedule, patient record (specifically the x-ray and progress notes), and the patient. These sources served as backups to one another, as illustrated by Dentist 7 when treating a patient for root canal. *"(The schedule) is where we start. I check the dental record to make sure the schedule is correct. Further back up is I always re-examine that particular tooth to see if, now, do I continue to agree with myself for what I wrote the last time they were here?" *Extra caution was exercised when special situations arose, such as when a patient came for reasons different from the schedule or had serious problems that required multiple procedures. In these cases, dentists sometimes confirmed with additional sources, such as an assistant or an outside specialist.

In summary, the unmet information needs of these dentists center on the quality and delivery of clinical information, reflected in their needs for complete, accurate and consistent information in a more timely fashion, presented in various formats with the best quality. In the general absence of online resources, these dentists meet their needs by using various sources based on the nature and complexity of problems and through cross-referencing to assure the accuracy and validity of information.

## Discussion

Dentists in the study reported a variety of information needs, with inquiries on chief complaints and symptoms, treatment options and procedures and parameters of problems at the top of the list. Regarding medical issues, dentists were most concerned about the impact of patients' pre-existing medical and health conditions (e.g., hypertension and diabetes) on their dental problems, especially duringon the use of local anesthesia. It appears that the rising prevalence of chronic diseases challenges general dentists in their understanding of oral-systemic interactions and the impact of multiple drug regimens. The knowledge of these information needs can inform the design of a dental information system, such as an electronic dental record (EDR). For example, in this study, dentists frequently searched for information on previous treatments and procedures, such as the length of files in a root canal case, in the progress notes section of the patient records. Therefore, progress notes could be highlighted and placed in a more prominent position in the layout of the EDR so that during a subsequent visit dentists can access previous treatment information on the first screen without having to search for them.

Although most of the dentists' questions were answered to their satisfaction, some information needs were not met or were only partially met. While most dentists used paper-based and electronic records simultaneously, the dual system sometimes led to incomplete, inconsistent and fragmented patient information. Clinicians routinely used x-rays to help diagnose patients' problems, but often the image quality was suboptimal. As a result, they desired quicker access to comprehensive and consistent patient information and better representation of dental problems in various formats. These together highlight the need for developing consolidated and comprehensive patient record systems that not only enable easier and more complete documentation but also include 2D or even 3D functionalities to better visualize patient problems.

In this study, some dentists desired more evidence-based information. A few dentists were trying to adopt an Evidence-Based Dentistry (EBD) treatment approach, but felt insufficient support due to the lack of evidence-based information accessible at the point of care, such as key statistics and clinical practice guidelines. This is supported by the Hannes and colleagues' study, which reported a lack of up-to-date evidence for devices and products and the complexity of guidelines regarding treatment choices as two major barriers to an EBD approach [36]. While The Cochrane Collaboration's Oral Health Group's Website http://www.ohg.cochrane.org/reviews.html lists 91 published systematic reviews of dental treatments and interventions as of August 2009, there are only three clinical recommendations on the ADA's EBD Website http://ebd.ada.org. Therefore, one focus of dental information systems could be on designing tools that help search for the most up-to-date clinical evidence and guidelines that match patient characteristics and better integrate this information into dental records in user-friendly way.

Getting quicker access to information, especially preauthorization by insurance companies, has also become a pressing need for dentists. Examples in this study show that patient care was sometimes unnecessarily delayed by the slow preauthorization process required by insurance companies. Policies that help reduce the authorization processing time would greatly benefit patients by speeding up treatment.

With respect to their unmet information needs, study participants displayed a somewhat ambivalent attitude. On one hand, they expressed frustration at not being able to get the information they needed; on the other, they tended to justify or even defend the unavailability of the information. They needed more evidence-based information, yet some had suspicions about the validity of research studies; they wanted to completely switch over to electronic patient records, yet harbored serious doubts about the authenticity and safety of computer data; they called for more cooperation and compliance from patients, yet sometimes did not fully trust what the patients said.

As for use of information sources, these dentists turned to a limited number of them for most questions. While the well-designated sections for information recording in patient records and the clear division of work in dental offices may explain the generally good match between information needs and sources, this by no means suggests that dentists are getting information in the best and most efficient way. It could be that dentists' use has adapted to the sources based on their availability and quality. Our analysis also showed that the nature of the patient problem affected the choice of sources, with the general rule being that as the level of complexity went up, the number of sources also increased, though with limited variability.

A notable source use pattern was that dentists routinely cross-referenced information to verify accuracy. This practice of information "triangulation," most commonly with the schedule, patient record and patients themselves, helped to prevent and correct errors in documentation and ensure that the dentist made the best clinical decision and performed the correct procedure. Always checking with the patient in the operatory also enabled the dentists to modify decisions based on the most up-to-date patient-related information available.

Although electronic sources are intended to facilitate and expedite information seeking, they did not play a prominent role in helping meet dentists' information needs during patient visits. A main reason seemed to be their fast-paced schedules and brief time with each patient, supplemented by the fact that when dentists did need and could search for information online, they failed to take action due to uncertainty about the availability of certain information, such as evidence-based information. As always, consulting with a colleague or specialist remained the first choice, however inefficient or disruptive this might be at times.

Putting the study results into context, it is apparent that dentists in this study have less information needs compared with their physician counterparts, as demonstrated by the number and scope of questions in Ely and colleagues' taxonomy of clinical questions [9]. While the methods used (interviews with a small sample versus surveys with a large sample) may explain some of the differences, we believe that the differences are more attributable to the fact that general dentists treat a relatively smaller number of clinical conditions in comparison to primary care physicians. However, our study identified the information that general dentists actually needed while seeing a patient instead of general information needs reported on a survey. We believe this approach, based on a user-centered design principle recommended by Kushniruk and Patel [37], will better inform the design of dental and clinical information systems.

## Limitations

This study has several limitations. First, we interviewed a convenience sample of dentists in Pittsburgh who volunteered to participate in the study. They might be systematically different from dentists who did not choose to participate in terms of information needs and source use. As a result, possible selection bias may exist, with this having an unknown impact on the study results. Therefore, caution needs to be taken when generalizing the results to other general dentists. Second, previous research has suggested that the office interview method might incur potential biases in two ways. On one hand, the interview may stimulate the dentists to think of more questions for our interviewer that were not there if they were not asked [38], on the other hand, dentists may report fewer information needs than they normally would due to their reluctance to reveal ignorance in front of the interviewer [39]. However, we are less concerned about the first bias as raising new questions about a patient in retrospection can still be helpful as long as they are important and relevant to the patient's diagnosis and treatment. Nonetheless, comparing the results with studies using different methodologies can help shed some light on the extent of the biases. Future studies can also use direct observations of treatment sessions in dental offices to validate our results. Third, given the fast-paced schedule of dentists, it is difficult to conduct in-depth interviews in dental offices; while we tried to follow up with the most crucial questions on the dentists' information needs and source use, time was not sufficient for the dentists to elaborate more on some of their answers.

## Conclusions

A challenge for designing useful clinical information systems in dentistry is to incorporate evidence-based information based on dentists' information needs and integrate the system seamlessly into the complex clinical workflow. As a critical step, we need to identify dentists' information needs and source use patterns. This study reveals that general dentists need a variety of information at the point of care, encompassing the medical aspects of dental problems and other issues affecting the condition. For optimal patient outcomes, quicker access to comprehensive patient and insurance information, better visual representation of patient problems, and access to key evidence-based guidelines are required. These findings can be used to inform the design of dental informatics tools. For example, the development of a comprehensive but easily accessible electronic dental record integrated with high-quality, up-to-date clinical evidence and 2D or 3D visual representation capabilities is a promising direction.

The study suggests limited variability among this group of dentists in the information sources used for each patient, but the type and number of sources in general correlates with the nature and complexity of the problem being treated. While the patient record and support staff remained the most used sources, electronic sources seem largely absent during patient visits. Triangulating sources for information verification is shown to be a standard practice for most dentists. Based on these findings, it is important to take into account the nature of dental problems when designing clinical information systems. For instance, these systems can support source triangulation for dentists by prompting them to consider using certain sources under specific clinical situations.

This study also demonstrates the usefulness of qualitative methods in providing an in-depth understanding of the issues and challenges related to dentists' information needs and source use in clinical settings. Similar techniques can be used to investigate information needs of other members of the profession such as dental specialists and hygienists.

## Competing interests

The authors declare that they have no competing interests.

## Authors' contributions

HS, DP and TS helped to conceptualize the study. HS and DP participated in the design of the study. TW carried out the original data collection. MS and HS performed the coding and thematic analysis. MS wrote the initial draft of the manuscript, which all authors reviewed and provided feedback on. All authors read and approved the final manuscript.

## Pre-publication history

The pre-publication history for this paper can be accessed here:

http://www.biomedcentral.com/1472-6947/10/7/prepub

## Supplementary Material

Additional file 1 **Post-patient interview of dentists**. This file includes the introduction to and questions of the interviews conducted with dentists after patient treatment sessions. Click here for file
