# Supplementary material for: How information systems should support the information needs of general dentists in clinical settings: suggestions from a qualitative study
Source: BMC Med Inform Decis Mak. 2010 Feb 2;10:7. doi: 10.1186/1472-6947-10-7 (PMC2843644; doi:10.1186/1472-6947-10-7)
Supplement: Additional file 1 — Post-patient interview of dentists. This file includes the introduction to and questions of the interviews conducted with dentists after patient treatment sessions. [file 1472-6947-10-7-S1.DOC]

# Additional file 1: Post-Patient Interview of Dentists

Introduction to dentists

The purpose of our interview with you today is to help us determine what dentists actually need to know while treating patients and how they use patient information sources, such as patient charts, to get the information they need.

We are interested in everything from a clear cut question (e.g., Does the patient have diabetes?) to the vague, fleeting uncertainties that you and I would normally keep to ourselves (e.g., I'm not totally sure why this patient is experiencing pain. It could be a hairline fracture or sensitivity. I’m going to call it sensitivity for now). Normally, we spend the day trying to convince our patients and our dental hygienists that we know what we're doing. I'm asking you to reveal your ignorance (to me—not to your patients or dental hygienist), which is not a natural thing to do. We’re looking for all the questions that come up during the patient session.

Here is what I’d like to do today:

I will stand in the hallway or your office and briefly talk to you after each patient you see. I will ask you about what happened during your time with the patient. I’m specifically interested in the information you need during your time with the patient. I’ll ask you about the questions that occurred to you and whether you were able to get the information you needed to answer the question. One thing is that I do NOT want you to say the name of the patient. This is important to keep your patients’ health information private.

For example, I’ll be interested in knowing what information you need and what information sources you used to get the information, such as the patient charts (including radiographs, images, etc.); what questions you asked the patient and support staff in the dental office, such as hygienist, dental assistant, reception desk, billing; and things that would have been more helpful to you that you did not know.

Post-patient interview questions

1. So, what was this patient visit about? Please don’t say the patient’s name.

2. What information (if any) did you look up in the patient chart (including radiographs, images, etc.)? Did you get the information you need or not?

3. What questions did you ask the patient?

4. Did you talk to people in the lab, other physicians, hygienist, dental assistant, reception desk or billing? What did you talk about?

5. If there was one more thing it would have been helpful to know, what would that have been?

Questions above are the basic questions; when necessary and appropriate, the researcher will follow up with the following questions:

- Talk me through the visit. What happened first? And then what? What were you thinking at that point? How did you decide to do that next? Was that helpful?
- So, you looked at the chart at X, Y, and Z. Did you look at the chart at any other time?
- What other sources did you use to get what you need?
- During the visit, did you ever have any questions about how best to care for the patient? At this point, do you have any questions about how best to care for the patient?
- Did the patient ask you any questions? What were the questions?
- Was there anything that would have been helpful to know that you didn’t know?
